# Peer support workers as a tool to expand access and improve the quality of care for transgender women and travestis in Brazil

**DOI:** 10.1590/S2237-96222024v33e2024324.especial.en

**Published:** 2024-12-16

**Authors:** Jae Sevelius, Gustavo Saggese, Jose Luis Gomez, Paula Galdino Cardin de Carvalho, Clair Aparecida da Silva Santos, Millena das Mercês de Oliveira Wanzeller, Sheri Lippman, Maria Amelia Veras

**Affiliations:** 1 Columbia University, Departament of Psychiatry, Nova Iorque, Estados Unidos; 2 Faculdade de Ciências Médicas da Santa Casa de São Paulo, Núcleo de Pesquisa em Direitos Humanos e Saúde LGBT+, SP, Brazil; 3 Faculdade de Ciências Médicas da Santa Casa de São Paulo, Departamento de Saúde Coletiva, SP, Brazil; 4 University of California San Francisco, School of Medicine, San Francisco, United States

Together we celebrate twenty years of transgender visibility in Brazil. Visibility can empower transgender people and their allies and encourage them to advocate for rights and protections, including legal recognition of gender identity, access to healthcare, and protection against discrimination.^1^ Unfortunately, much work remains to be done in these areas. Globally, transgender women face extreme vulnerability and marginalization, including in Brazil.^2^ Experiences of stigma and discrimination lead to social isolation and violence; regrettably, the country is often cited as having some of the highest rates of transphobic violence in the world.^3^

Experiences of discrimination and violence increase susceptibility to negative health outcomes, including HIV and poor mental health.^4^–^6^ Rates of depression, anxiety, substance use, trauma symptoms, and suicidal ideation are higher among trans women compared to the general population.^7,8^ Thus, the demand for healthcare services is consistently high; however this population often avoid health services due to anticipated and experienced stigma and discrimination.^9^ Experiences of stigma, discrimination, and violence are even more common among transgender women facing additional oppressions, such as racism and classism.

In Brazil, the Psychosocial Care Network (*Rede de Atenção Psicossocial* – RAPS), which includes Psychosocial Care Centers, aims to provide free mental health services to individuals suffering intensely from psychological disorders, substance abuse and experiencing various crises. RAPS often fails to adequately serve individuals with non-acute mental health symptoms – such as mild to moderate depression and anxiety,^10^ which are common among transgender people – associated with unmeet gender affirming needs and chronic invalidation of their identities.

The shortage of human resources, particularly in mental health, demands alternative strategies to bridge existing gaps in care for others. Peer support interventions have proven invaluable across many health domains, including mental health, cancer, substance use, and HIV treatment and prevention, demonstrating success in addressing support and care needs among vulnerable populations.^11-13^ These interventions are crucial in building relationships that promote behavior change and can effectively address trauma symptoms and develop coping and resilience skills in the face of stigma, improve social gender affirmation, and help prevent the internalization of stigmatizing experiences.^14^

Our team has collectively worked in Brazil for many years developing and testing peer support interventions for travestis and transgender women. These interventions have shown feasibility, acceptability, and effectiveness in reducing symptoms of trauma, depression, anxiety, and stress, while enhancing coping skills, and increasing acceptance of HIV prevention and treatment.^15-18^ This approach is gaining popularity because it can provide health benefits to both participants and peers; promote empowerment; maximize acceptability; increase access for marginalized groups; facilitate dissemination of these actions; and reduce intervention costs.^11^

Peer support can increase individuals’ feelings of being seen and understood, which can positively impact mental health and well-being among transgender people by reducing feelings of isolation and stigma.^14^ Peer support increases resilience to stigma and reduces internalized stigma, and people who receive it often report less psychological distress caused by stigma experiences. They are also more likely to engage in health care and increase self-care behaviors, leading to better health outcomes.^19^

## REPORT OF OUR EXPERIENCE

In our studies conducted in São Paulo and the United States, transgender people enthusiastically expressed a desire for peer support (referred to as peer navigation) from those who understand their experiences and the challenges of engaging in health care. Peer navigators provide practical assistance within healthcare systems – including how to handle experiences of stigma and discrimination – as well as urgently needed emotional support informed by shared experiences and that foster mutual understanding, respect, and empowerment. Unlike professional power dynamics, peer support is a “give-and-receive system,” guided by core principles such as shared responsibility and mutual agreement on what is considered helpful.^20^ These type of support differs from friendship as the focus of the relationship is to help the recipient obtain and understand relevant information to set goals and make the best choices for themselves. It also differs from health care provision: once goals are collaboratively established, hierarchies are avoided and shared lived experience is encouraged.

In the interventions we have developed in collaboration with travestis and transgender women, peer navigators engage in various activities. Important tasks include familiarizing themselves with healthcare and social assistance networks, and legal aid resources, providing referrals or accompanying individuals to access these services. Peer navigators also offer basic information, share personal experiences, strengthen and expand support networks, facilitate groups, develop skills, provide individual guidance, and help set personalized goals with practical steps. To effectively perform these activities, peer workers require ongoing training and supervision to ensure they engage with participants carefully and collaboratively, maintaining personal and professional boundaries, which can be challenging within community settings.

Peer support workers share many lived experiences with those they support, making the transition into this role potentially challenging.^21^ These workers require compassionate guidance from trained supervisors to monitor and maintain their own mental health, avoiding secondary traumatic stress – emotional distress resulting from exposure to the trauma experienced by the participants they support.. In Brazil, we found it essential to provide regular supervision to these workers to ensure they have the guidance needed to navigate the challenging situations that inevitably arise and to succeed in their role.

In addition to the direct benefits to peers and recipients, the visibility of peer support workers in the healthcare system can lead to greater social inclusion and awareness, as well as a better understanding of the issues faced by transgender people, including the need for inclusive policies and practices. Seeing transgender people various roles and positions in society helps diversify their representations and challenges stereotypes and misconceptions. The visibility of transgender people serving as peer support workers in the healthcare system can validate their experiences and identities, affirming that they are valued members of society with unique needs, perspectives, and contributions.

Despite the benefits, introducing peer support services into the Brazilian health system can pose challenges, as systemic change can be slow and resources are often scarce.^11^ In healthcare settings, barriers to integrating peer support workers often include confusion about their role, resistance from other team members, and unequal treatment of peer support workers, including stigmatization.^21^ Clear definition of the role, personal and supervisory training, and sufficient ongoing support should be carefully considered and provided.

## FUTURE PERSPECTIVES

Although there is extensive empirical evidence supporting the benefits of peer support in improving health and well-being among transgender people, research on the successful implementation and scaling of peer support remains in its infancy and urgently needed. Many scholars have advocated for systematic research on the processes involved in the implementation of peer-led health promotion programs and the conductive conditions for effectively integrating this workforce into health systems, including the necessary systematic changes to accommodate such programming.^11,14,15,22^ Understanding the conditions for implementation is crucial to determining the facilitators and barriers to effective peer support services in Brazil, as well as to identifying the optimal contexts where these programs can yield the greatest benefits and the policies needed to ensure sustainability of these programs.

Peer support workers can play a critical role in serving transgender people in Brazil, where stigma and discrimination often lead to poor-quality care, avoidance of seeking care, and many unmet health needs within transgender communities, especially among those facing intersectional oppressions, such as Black travestis and transgender women and those living with HIV. Peer support aligns well with the principles of making public health services more human-centered, a stated commitment of the Brazilian National Health System.^23^ Using peer support helps identify and address health needs from social, collective, and personal perspectives. This form of support also respects people from different communities involved in healthcare and encourages their independence and active participation. We believe that by working with the community, we can drive the systemic changes needed to create conditions where we can thrive and lead healthier lives.
