# Supplementary material for: Peer support workers as a tool to expand access and improve the quality of care for transgender women and travestis in Brazil
Source: Epidemiol Serv Saude. 2024 Dec 16;33(spe1):e2024324. doi: 10.1590/S2237-96222024v33e2024324.especial.en (PMC11654814; doi:10.1590/S2237-96222024v33e2024324.especial.en)
Supplement: Translation

# Translation: O trabalho de apoio de pares como ferramenta para ampliar o acesso e a qualidade do cuidado para mulheres trans e travestis no Brasil

**DOI:** 10.1590/S2237-96222024v33e2024324.especial.pt

**Authors:** Jae Sevelius, Gustavo Saggese, Jose Luis Gomez, Paula Galdino Cardin de Carvalho, Clair Aparecida da Silva Santos, Millena das Mercês de Oliveira Wanzeller, Sheri Lippman, Maria Amelia Veras

**Affiliations:** 1 Columbia University, Departament of Psychiatry, Nova Iorque, Estados Unidos; 2 Faculdade de Ciências Médicas da Santa Casa de São Paulo, Núcleo de Pesquisa em Direitos Humanos e Saúde LGBT+, SP, Brasil; 3 Faculdade de Ciências Médicas da Santa Casa de São Paulo, Departamento de Saúde Coletiva, SP, Brasil; 4 University of California San Francisco, School of Medicine, San Francisco, Estados Unidos

Juntas, comemoramos vinte anos de visibilidade trans no Brasil. A visibilidade pode empoderar pessoas trans e suas aliadas e incentivá-las a defender direitos e proteções, incluindo o reconhecimento legal da identidade de gênero, o acesso a cuidados de saúde e a proteção contra discriminação.^1^ Infelizmente, ainda há muito trabalho a fazer nesses domínios. Globalmente, travestis e mulheres trans enfrentam extrema vulnerabilidade e marginalização, inclusive no Brasil.^2^ Experiências de estigma e discriminação levam ao isolamento social e à violência; de maneira lamentável, o país é frequentemente citado como tendo alguns dos maiores índices de violência transfóbica do mundo.^3^

Experiências de discriminação e violência aumentam a suscetibilidade a resultados negativos de saúde, os quais abrangem HIV e saúde mental precária.^4-6^ As taxas de depressão, ansiedade, uso de substâncias, sintomas de trauma e ideação suicida são mais altas entre mulheres trans quando comparadas com a população em geral.^7,8^ Assim, as demandas por cuidados de saúde são constantemente elevadas; porém, esse coletivo muitas vezes evita os serviços de saúde devido ao estigma e à discriminação antecipados e já vivenciados.^9^ As experiências de estigma, discriminação e violência são ainda mais comuns entre travestis e mulheres trans que experimentam opressões adicionais, como racismo e classismo.

No Brasil, a Rede de Atenção Psicossocial (RAPS), que engloba os Centros de Atenção Psicossocial, tem como objetivo oferecer serviços gratuitos de saúde mental para os indivíduos que sofrem, de modo intenso, em decorrência de transtornos psicológicos, abusam de substâncias e vivenciam crises diversas. A RAPS muitas vezes não atende adequadamente os indivíduos com sintomas de saúde mental não agudos – como depressão leve a moderada e ansiedade,^10^ comuns entre pessoas trans –, associados ao não atendimento de suas necessidades de afirmação de gênero e à invalidação crônica de suas identidades. 

A escassez de recursos humanos, particularmente em saúde mental, demanda estratégias alternativas para minimizar as lacunas existentes no cuidado com o outro. As intervenções de apoio entre pares provaram ser inestimáveis em muitos domínios da saúde, incluindo saúde mental, câncer, uso de substâncias e tratamento e prevenção do HIV, e mostraram-se bem-sucedidas na abordagem de necessidades de apoio e cuidado entre populações vulneráveis.^11-13^ Essas intervenções são fundamentais na construção de relacionamentos que favoreçam mudanças de comportamento e podem efetivamente abordar os sintomas do trauma e desenvolver habilidades para o enfrentamento e a resiliência ao estigma, melhorar a afirmação social de gênero, e ajudar a evitar a internalização de experiências estigmatizantes.^14^

Coletivamente, nossa equipe tem trabalhado no Brasil por muitos anos desenvolvendo e testando intervenções de apoio entre pares para travestis e mulheres trans. Tais intervenções demonstraram viabilidade, aceitabilidade e efetividade na redução de sintomas de trauma, depressão, ansiedade e estresse, ampliando o desenvolvimento de habilidades de enfrentamento e aumentando a aceitação da prevenção e do tratamento do HIV.^15-18^ Esse procedimento está crescendo em popularidade porque pode proporcionar benefícios à saúde para participantes e pares; promover o empoderamento; maximizar a aceitabilidade; aumentar o acesso para grupos marginalizados; facilitar a disseminação dessas ações; e reduzir os custos da intervenção.^11^

O apoio dos pares pode aumentar os sentimentos do indivíduo de ser visto e compreendido, o que pode impactar positivamente a saúde mental e o bem-estar entre as pessoas trans, reduzindo os sentimentos de isolamento e estigma.^14^ O apoio dos pares aumenta a resiliência ao estigma e reduz o estigma internalizado, e as pessoas que o recebem frequentemente relatam menos sofrimento psicológico causado por experiências de estigma. Elas também são mais propensas a cuidar da saúde e aumentar os comportamentos de autocuidado, que levam a melhores resultados de saúde.^19^

## RELATO DA NOSSA EXPERIÊNCIA

Em nossos estudos realizados em São Paulo e nos Estados Unidos, travestis e mulheres trans expressaram entusiasticamente o desejo de receber apoio de pares (chamamos de navegação por pares) que entendam suas experiências e os desafios em se engajar aos cuidados de saúde. Navegadoras de pares fornecem apoio no acesso aos sistemas de cuidados de saúde – o que inclui como lidar com experiências de estigma e discriminação –, bem como apoio emocional urgentemente necessário, o qual é informado por experiências compartilhadas, e que geram compreensão mútua, respeito e empoderamento. Não baseado em uma dinâmica de poder profissional, o apoio entre pares é um “sistema de dar e receber ajuda”, guiado por princípios fundamentais como responsabilidade compartilhada e acordo mútuo sobre o que é considerado útil.^20^ Tal apoio difere de uma amizade, pois o foco da relação é ajudar o receptor do apoio a obter e compreender informações relevantes para definir metas e fazer as melhores escolhas para si mesmo. Também difere da prestação de cuidados de saúde: uma vez que as metas são estabelecidas de forma colaborativa, as hierarquias são evitadas e a experiência de vida compartilhada é incentivada.

Nas intervenções que desenvolvemos em colaboração com travestis e mulheres trans, as navegadoras de pares se envolvem em vários tipos de atividades. Tarefas importantes incluem conhecer as redes de saúde e de assistência social, recursos de assistência jurídica, para poder articular e/ou acompanhar as pessoas na obtenção desses serviços. As navegadoras de pares também fornecem informações básicas, compartilham experiências pessoais, fortalecem e ampliam as redes de apoio, facilitam grupos, desenvolvem habilidades, fornecem orientação individual e ajudam a definir metas individualizadas e com tarefas práticas. Para executar com competência essas atividades, tais trabalhadoras precisam de treinamento e supervisão continuada para garantir que estejam se envolvendo com as participantes de forma cuidadosa e colaborativa, mantendo os limites pessoais e profissionais, o que pode ser desafiador no contexto de uma comunidade. 

As trabalhadoras de apoio entre pares compartilham muitas experiências vividas com as pessoas que estão apoiando, por isso a transição para trabalhar nessa área pode ser desafiadora.^21^ Essas trabalhadoras precisam de orientação compassiva de uma supervisão treinada para monitorar e manter sua própria saúde mental, evitando o estresse traumático secundário – sofrimento emocional que pode ser resultado da exposição ao trauma experimentado pelas participantes a quem estão apoiando. No Brasil, descobrimos que é fundamental fornecer supervisão regular a tais trabalhadoras para garantir que estas tenham a orientação de que precisam para navegar as situações desafiadoras que inevitavelmente surgem e para ser bem-sucedidas em seu papel.

Além dos benefícios diretos para pares e receptores, a visibilidade das trabalhadoras de apoio entre pares no sistema de saúde pode levar a uma maior inclusão social e conscientização, bem como à compreensão dos problemas enfrentados pelas pessoas trans, incluindo a necessidade de políticas e práticas inclusivas. Ver pessoas trans em vários papéis e posições na sociedade ajuda a diversificar as representações dessas pessoas e desafia estereótipos e equívocos. A visibilidade de travestis e pessoas trans atuando em papéis de apoio entre pares no sistema de saúde pode validar suas experiências e identidades, afirmando que são membros valorizados da sociedade com necessidades, perspectivas e contribuições únicas.

Apesar dos benefícios, a introdução de serviços de apoio entre pares no sistema de saúde brasileiro pode representar desafios, uma vez que mudanças sistêmicas podem ser lentas, e os recursos, muitas vezes, escassos.^11^ Em ambientes de cuidados de saúde, os obstáculos à integração de trabalhadoras de apoio entre pares geralmente incluem confusão sobre o papel destas, resistência de outros membros da equipe e tratamento desigual de trabalhadoras de apoio entre pares, incluindo estigmatização.^21^ A clareza sobre o papel de quem trabalha nessa área, a formação pessoal e da supervisão e o apoio suficiente e continuado devem ser cuidadosamente considerados e fornecidos.

## PERSPECTIVAS FUTURAS

Embora haja amplos dados empíricos sobre o benefício do apoio entre pares para melhorar a saúde e o bem-estar entre pessoas trans, a pesquisa sobre a implementação e a ampliação bem-sucedidas de serviços de apoio entre pares é ainda incipiente e urgentemente necessária. Muitos estudiosos têm defendido pesquisas sistemáticas sobre o processo envolvido na implementação de programas de promoção da saúde liderados por pares e condições propícias à integração efetiva dessa força de trabalho nos sistemas de saúde, o que inclui mudanças necessárias nos sistemas para acomodar tal programação.^11,14,15,22^ Compreender as condições de implementação é crucial para determinar os facilitadores e as barreiras para serviços efetivos de apoio entre pares no Brasil, bem como para identificar os contextos ótimos onde esses programas podem produzir o máximo de benefícios e políticas necessárias com o intuito de garantir que os programas sejam sustentados. 

O trabalho de apoio entre pares pode desempenhar um papel crítico no atendimento a pessoas trans no Brasil, no qual o estigma e a discriminação muitas vezes levam a cuidados de baixa qualidade, evitação da busca por assistência e muitas necessidades não atendidas de cuidados entre as comunidades trans, especialmente aquelas que enfrentam opressões interseccionais, como mulheres trans negras e aquelas que vivem com HIV. O apoio entre pares se alinha bem com os princípios de tornar os serviços públicos de saúde mais centrados no ser humano, um compromisso declarado do Sistema Único de Saúde.^23^ A utilização do apoio de pares ajuda a identificar e abordar as necessidades de saúde a partir de perspectivas sociais, coletivas e pessoais. Esse apoio também respeita as pessoas de diferentes comunidades envolvidas na área da saúde e incentiva a sua independência e participação ativa. Acreditamos que podemos trabalhar com a comunidade para realizar as mudanças sistêmicas de que precisamos, com a finalidade de criar as condições de prosperar e viver nossas vidas de modo mais saudável.
